# Density Gradient Centrifugation Compromises Bone Marrow Mononuclear Cell Yield

**DOI:** 10.1371/journal.pone.0050293

**Published:** 2012-12-06

**Authors:** Claudia Pösel, Karoline Möller, Wenke Fröhlich, Isabell Schulz, Johannes Boltze, Daniel-Christoph Wagner

**Affiliations:** 1 Fraunhofer Institute for Cell Therapy and Immunology, Leipzig, Germany; 2 Translational Centre for Regenerative Medicine, Leipzig, Germany; Foundation for Applied Medical research, Spain

## Abstract

Bone marrow mononuclear cells (BMNCs) are widely used in regenerative medicine, but recent data suggests that the isolation of BMNCs by commonly used Ficoll-Paque density gradient centrifugation (DGC) causes significant cell loss and influences graft function. The objective of this study was to determine in an animal study whether and how Ficoll-Paque DGC affects the yield and composition of BMNCs compared to alternative isolation methods such as adjusted Percoll DGC or immunomagnetic separation of polymorphonuclear cells (PMNs). Each isolation procedure was confounded by a significant loss of BMNCs that was maximal after Ficoll-Paque DGC, moderate after adjusted Percoll DGC and least after immunomagnetic PMN depletion (25.6±5.8%, 51.5±2.3 and 72.3±6.7% recovery of total BMNCs in lysed bone marrow). Interestingly, proportions of BMNC subpopulations resembled those of lysed bone marrow indicating symmetric BMNC loss independent from the isolation protocol. Hematopoietic stem cell (HSC) content, determined by colony-forming units for granulocytes-macrophages (CFU-GM), was significantly reduced after Ficoll-Paque DGC compared to Percoll DGC and immunomagnetic PMN depletion. Finally, in a proof-of-concept study, we successfully applied the protocol for BMNC isolation by immunodepletion to fresh human bone marrow aspirates. Our findings indicate that the common method to isolate BMNCs in both preclinical and clinical research can be considerably improved by replacing Ficoll-Paque DGC with adapted Percoll DGC, or particularly by immunodepletion of PMNs.

## Introduction

Bone marrow transplantation was originally established to treat hematological malignancies [1] and is nowadays widely used in different branches of regenerative medicine. The bone marrow is a capable source of autologous cells with distinct regenerative properties, which can be quickly harvested and are thus applicable for both chronic and acute diseases. Preclinical and clinical safety, feasibility and efficacy have been reported, inter alia, for ischemic limb injury [2], [3], cerebral ischemia [4], [5] and in particular for myocardial infarction [6], [7] for which by now more than 30 placebo controlled randomized trials have been accomplished [8].

In the majority of studies, aspirated bone marrow was further processed in order to isolate the mononuclear cell fraction (BMNC), a heterogeneous population containing differentially matured B-cells, T-cells and monocytes, as well as rare progenitor cells such as hematopoietic stem cells (HSC), mesenchymal stromal cells (MSC), endothelial progenitor cells (EPC) and very small embryonic-like cells (VSEL). It has been constantly described that this cell mixture promotes distinct angiogenic properties [2], mediates vascular repair, expresses several cytoprotective growth factors and cytokines [9] and restores pathologically altered genes after ischemic heart injury [10]. However, which component or combination of components exactly determines the efficacy of BMNCs is not entirely understood, hence impeding full realization and further advancement of the therapeutic concept [11]. Some groups suggested that conflicting results in large-scale clinical trials [12], [13] are, at least to some extent, due to different cell isolation protocols and a subsequently altered BMNC composition [14]. In fact, it has been proven that efficacy and functionality of BMNCs are significantly influenced by red blood cell contamination [15], the content of apoptotic cells [16], different washing steps [14] and even by the centrifugation speed [17].

Another decisive point seems to be the choice of the density gradient medium. Most preclinical and clinical studies used Ficoll-Paque (hereafter indicated as Ficoll) as density medium in order to enrich the mononuclear cell population as well as the rare progenitor cells therein [18]. However, it is a well-known problem that Ficoll-based density gradient centrifugation (DGC) causes a significant reduction of BMNCs to only 15–30% of the initial content [17], [19]. This is critical since the efficacy of autologous BMNC transplantation is likely dose-dependent [20], and little data is available on a possible asymmetry of the cell loss [21], [22]. Recently, it was described that Ficoll DGC even depleted cells with a high regenerative potential, such as MSC [23] and VSEL [24], and irreversibly impaired cell function by decreasing expression of chemokines receptors [25], [26]. Accordingly, the objective of this study was to determine whether and how Ficoll DGC affects the yield and composition of the cell graft compared to alternative methods such as adjusted Percoll DGC [27] and immunomagnetic bead separation of granulocytes [28]. Our findings indicate that the common method to isolate BMNC in both preclinical and clinical research can be considerably improved by replacing Ficoll with adapted Percoll or preferably by immunodepletion of unwanted constituents of bone marrow.

## Methods

### Rat Bone Marrow Harvest and Lysis of Erythroid Cells

Animal experiments were conducted according to the Guide for the Care and Use of Laboratory Animals published by the US National Institutes of Health (NIH Publication No. 85-23, revised 1996). Rat bone marrow was obtained from 12-week-old male Sprague-Dawley rats. Femurs and tibiae were aseptically opened and bone marrow was harvested by repeated flushing with phosphate buffered saline (PBS). In order to dissolve remaining cell aggregates, the solution was resuspended using a 20 G cannula and sieved through a 100 µm cell strainer. Erythroid bone marrow cells were lysed by short-term incubation (30 seconds) with hypotonic ammonium chloride buffer (0.155 M NH4Cl, 10 mM KHCO3 and 0.01 mM Na2EDTA) followed by repeated washing steps with PBS containing 3% fetal calf serum (PBS/3% FCS). Viability and cell count were determined by the trypan blue exclusion method using a hemocytometer.

### Rat Mononuclear Cell Isolation by Density Gradient Centrifugation

Bone marrow cells from 5 donor rats were separated using Ficoll-Paque 1.084 (which is used to isolate rat mononuclear cells (MNC; GE Healthcare, Munich, Germany) with 3 technical replicates per sample. A total of 10E7 bone marrow cells were resuspended in 10 mL HBSS/3% FCS and carefully layered upon 7.5 mL of Ficoll separation medium. Ficoll gradients were centrifuged for 40 min at 400 g without brake. The bone marrow mononuclear cell (BMNC) layer was then collected, washed in PBS/3% FCS, counted and prepared for flow cytometric analysis.

Next, we aimed to establish an appropriate density gradient for isolating BMNC using Percoll medium (GE Healthcare, Munich, Germany). Concentrated Percoll was diluted in 1.5 M NaCl to an isotonic Percoll stock solution (SIP) with a density of 1.1228 g/mL. We then prepared a series of different separation media (1.071, 1.073, 1.075, 1.077, 1.080 and 1.084 g/mL) by mixing SIP with varying volumes of Hank’s balanced salt solution (HBSS) containing 3% FCS. Likewise, an invariant bottom layer was adjusted to 1.095 g/mL. For each of the separation gradients, 10E7 bone marrow cells from two donor rats were resuspended in 5 ml Percoll medium with the highest density (1.095 g/mL). This layer was then carefully covered with 5 ml of the respective lower concentrated Percoll dilutions and finally capped with 5 mL of HBSS/3% FCS. Discontinuous gradients were centrifuged for 40 min at 480 g without brake. After centrifugation, the cell layers located between HBSS/separation medium or in between the two Percoll layers were separately collected and washed twice in PBS/3% FCS. Cells were counted and prepared for flow cytometric analysis. The Percoll medium density leading to the highest BMNC yield along with the lowest polymorphonuclear cells (PMN) contamination (1.080 g/mL) was then chosen for according BMNC isolation from 5 donor rats with 3 technical replicates per sample.

### Rat BMNC Isolation by Immunomagnetic Depletion of PMNs

A total of 10E8 bone marrow cells obtained from 6 donor rats (with 3 technical replicates per sample) were incubated with 5 ng/ml phycoerythrin conjugated anti-rat granulocytes antibody (clone RP1; BD Pharmingen, Heidelberg, Germany) for 15 min at 4°C. After washing in cold PBS/0.5% FCS, bone marrow cells were further incubated with 50 µL magnetic anti-phycoerythrin microbeads (Miltenyi Biotech, Bergisch Gladbach, Germany) for 12 min at 4°C. Non-adsorbed microbeads were eliminated by an additional washing step. Finally, bone marrow cells were resuspended in 500 µL PBS/0.5% FCS. Magnetic separation was performed on LD columns using a magnetic QuadroMACS separator according to manufacturer’s instructions (Miltenyi Biotech). Both discharge and the fraction that was magnetically trapped were separately collected, counted and prepared for flow cytometry.

### Flow Cytometric Characterization of rat BMNCs

**Figure 1:**
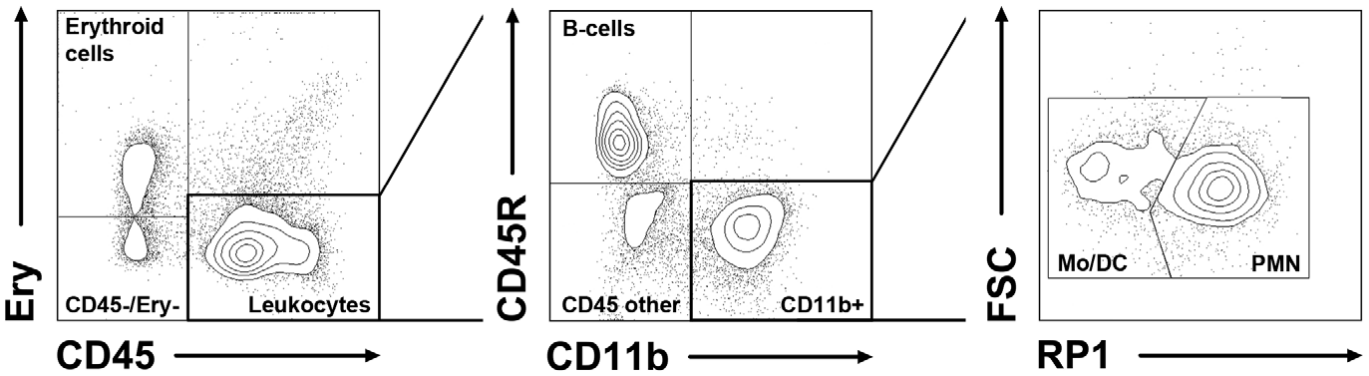
Representative illustration of the gating strategy in rat samples. The cells of interest were first categorized into cells belonging to the erythroid lineage (CD45−/Ery+), CD45−/Ery- cells and leukocytes (CD45+/Ery-). The latter were then differentiated into B-cells (CD45R+/CD11b−), CD11b+ cells (CD45+/CD45R−/CD11b+) and other CD45+ cells (CD45+/CD45R−/CD11b−). Next, CD11b+ cells were separated into RP1+ polymorphonuclear cells (PMN) and RP1- monocytes/dendritic cells (Mo/DC).

Cellular composition of lysed rat bone marrow and separated BMNCs following different isolation protocols were analyzed by flow cytometry. For labelling, 2.5×10E5 cells were incubated with a mixture of monoclonal antibodies (Table 1) for 20 min at 4°C. Erythroid cells were labeled by biotinylated anti-erythroid-antibody which was secondly conjugated with streptavidin PerCP (BD Pharmingen, Heidelberg, Germany). After incubation, cells were washed and resuspended in 300 µL PBS/3% FCS. Flow cytometric acquisition and analysis was performed using a FACS Canto II equipped with FACS Diva software (BD Biosciences, Heidelberg, Germany). Cellular subpopulations were identified by specific antigen expression (Table 1) and categorized according to the gating strategy displayed in Figure 1.

**Table 1:** Anti-rat monoclonal antibodies used for flow cytometry.

FC panel				Cell population					
Antigen	Fluorochrome	Clone	Manufacturer	B-cells	Mo/DC	PMNs	CD45 other	Erythroidcells	CD45−/Ery-
CD45R	FITC	HIS24	BD	+	−	−	−	−	−
Granulocytes	PE	RP-1	BD	−	−	+	−	−	−
Erythroid	Biotin	HIS49	BD	−	−	−	−	+	−
CD45	APC-Cy7	OX-1	BD	+	+	+	+	−	−
CD11b	Pacific Blue	MRC-OX42	Abd Serotec	−	+	+	−	−	−

### Determination of Progenitor Frequency in Rat Samples

The frequency of hematopoietic or non-hematopoietic progenitors was assessed by granulocyte-macrophage (CFU-GM) and fibroblast (CFU-F) colony forming unit assays. For CFU-GM, 1.5×10E4 vital cells were seeded in duplicates with 1.1 ml Methocult medium (GF R3774, Stem Cell technologies, Grenoble, France) into 35 mm suspension dishes and further cultivated at 37°C, 5% CO_2_ and 95% humidity for 14 days. Proliferating CFU-GM colonies were counted and the frequency was calculated and normalized to leukocytes counts. CFU-GM frequency was analyzed from 8 (lysed bone marrow) or 4 (Percoll, Ficoll and MACS) donor rats with 2 technical replicates per sample.

Accordingly, to determine CFU-F frequency, 10E7 vital cells from 8 (lysed bone marrow) or 4 (Percoll, Ficoll and MACS) donor rats (with 2 technical replicates per sample) were plated into 35 mm culture dishes in Dulbecco’s modified Eagle’s medium containing 4.5 g/L glucose (PAA Laboratories, Cölbe, Germany), 10% FCS plus 1% Penicillin/Streptomycin and maintained at 37°C, 5% CO_2_ and 95% humidity for 21 days. Medium was initially changed after 5 days and twice a week thereafter. After 3 weeks, cells were fixed in ice-cold methanol and stained with Giemsa. Fibroblast colonies were counted and frequencies were calculated.

### Pretreatment of Human Bone Marrow Aspirates

Next, we aimed to validate the protocol of BMNC isolation by immunodepletion in human samples. Human bone marrow aspirates from 3 donors were purchased from Lonza Walkersville (Lonza, Walkersville, MD; FDA-registered company for the processing of human cells, tissue and cellular and tissue based products in accordance with the US Code of Federal Regulations (21 CFR Par 1271)). Human bone marrow was diluted 10-fold with PBS/3% FCS and 5 mM EDTA and sieved through a 30 µm cell strainer (pluriSelect, Leipzig, Germany) to exclude fat and bone fragments. After centrifugation for 5 min at 440 g, human bone marrow was resuspended in PBS/3% FCS and 5 mM EDTA to the original volume.

Each of the three bone marrow samples was then split into two experimental groups: (i) whole human bone marrow and (ii) lysed human bone marrow. For lysis, human bone marrow cells were incubated with a 4 fold volume of hypotonic ammonium chloride buffer (0.155 M NH4Cl, 10 mM KHCO3 and 0.1 mM Na2EDTA) for 10 min at room temperature followed by repeated washing steps with PBS/3% FCS. Viability and total cell count were determined by the trypan blue exclusion method in a hemocytometer. Leukocytes were separately stained and counted with Turk’s solution.

### Immunodepletion of PMNs from Human Bone Marrow

First, PMNs were depleted from lysed human bone marrow by means of magnetic-activated cell sorting (MACS). One mL of lysed bone marrow was incubated with 50 µL CD15 whole blood MicroBeads (for human PMNs; Miltenyi Biotech) for 20 min at 4°C. Non-adsorbed MicroBeads were eliminated by washing with PBS/0.5% FCS. The labeled cell suspension was then resuspended in 2 mL of PBS/0.5% FCS. Subsequently, magnetic separation was performed on LS columns using a QuadroMACS separator according to the manufacturer’s instructions (Miltenyi Biotech). LS columns were successively loaded with 0.5 mL of CD15-labeled lysed bone marrow cells and 1 mL of PBS/0.5% FCS. Both discharge and the magnetically trapped CD15+ fraction were separately collected, counted and prepared for flow cytometry.

Alternatively, PMNs were depleted from lysed human bone marrow using the PluriBead technology (PluriB) that bases on differentially sized beads (rosetted with according antibodies) that can be separated by cell strainers (pluriStrainer, pluriSelect). One mL of lysed bone marrow was incubated with anti-human CD15 whole blood S-pluriBeads (pluriSelect; with a ratio of 2 pluriBeads per 3 target cells) on a pluriPlix shaker (pluriSelect) for 30 min at room temperature. Next, the labeled cell suspension was passed through an S-pluriStrainer (pluriSelect) accompanied by continuous rinsing with 15 mL of washing buffer (pluriSelect). Cells that were trapped in the S-pluriStrainer were detached from the pluriBeads using 1 mL detachment buffer (pluriSelect) for 10 minutes at room temperature and flushed through the strainer with 6 mL of washing buffer. Both fractions were separately collected, counted and prepared for flow cytometry.

In the next step, we considered to replace lysis by immunodepletion of erythrocytes. For immunomagnetic depletion of erythrocytes, whole human bone marrow was incubated with 300 µL of CD235a MicroBeads (Glycophorin A; for human erythroid cells; Miltenyi Biotech) for 20 min at 4°C. Further steps correspond to those described for the immunomagnetic CD15 depletion (above), except for the loading of the LS columns. Due to the significantly higher number of magnetically labeled cells (ratio of leucocytes to erythrocytes in whole bone marrow was 1∶200), 1 mL of CD235a-labeled whole bone marrow was loaded onto 3 LS columns. Both discharge and the mobilized CD235a+ cells were separately collected, counted and prepared for flow cytometry. In a second step, discharge was further immunomagnetically depleted of CD15+ PMNs as described above.

Finally, we used the pluriBead technology to combine multiple protocol steps by using differentially sized beads to simultaneously deplete erythrocytes and PMNs. Whole human bone marrow was incubated with anti-human CD235a M-pluriBeads (with a ratio of 1 M-pluriBead per 140 target cells) and CD15 whole blood S-pluriBeads (with a ratio of 2 pluriBeads per 3 target cells) for 30 min at room temperature on a pluriPlix shaker. Further steps correspond to those described for the CD15 pluriBead depletion (above).

### Flow Cytometric Characterization of Human BMNC

Cellular composition of human bone marrow cells and subsequently separated cell fractions were analyzed by means of flow cytometry according to the protocols described for the analysis of rat BMNCs (above). Human cellular subpopulations were identified by expression of specific antigens (Table 2).

**Table 2:** Anti-human monoclonal antibodies used for flow cytometry.

FC panel				Cell population						
Antigen	Fluorochrome	Clone	Manufacturer	B-cells	T-cells	Monocytes	PMNs	Erythroid cells	MSC	HSC
CD15	FITC	VIMC6	Miltenyi	−	−	−	+	−	N/A	N/A
CD3	PE	UCHT1	BD	−	+	−	−	−	N/A	N/A
CD235a	PE-Cy5	GA-R2 (HIR2)	BD	−	−	−	−	+	N/A	N/A
CD14	PE-Cy7	M5E2	BD	−	−	+	−	−	N/A	N/A
CD19	APC	HIB19	BD	+	−	−	−	−	N/A	N/A
CD45	APC-Cy7	2D1	BD	+	+	+	+	−	−	+
CD133	PE	AC133	Miltenyi	−	−	−	−	−	−	(+)
CD105	PerCPCy5.5	266	BD	−	−	−	−	−	+	−
CD34	APC	AC136	Miltenyi	−	−	−	−	−	−	+

### Statistical Analysis

Statistical differences were analyzed using t-tests (in case of 2 groups) or by one way analysis of variance (ANOVA; in case of more than 2 groups) and Holm-Sidak post hoc test. A p-value of 0.05 or less was considered statistically significant. All data was shown as mean ± standard deviation (SD).

## Results

### Rat Bone Marrow Cell Yield and Effect of Lysis

**Figure 2:**
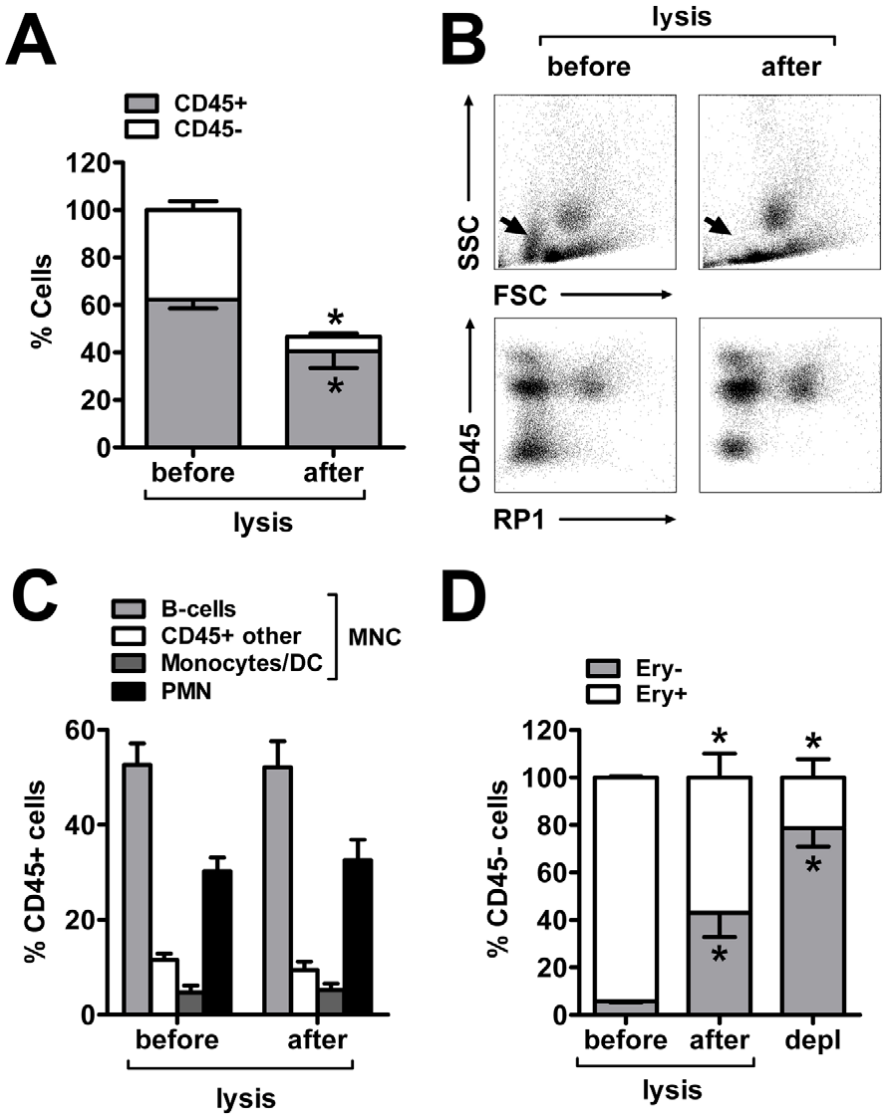
Influence of ammonium chloride lysis on rat bone marrow composition. (A) Freshly isolated rat bone marrow consists of 60% CD45+ and 40% CD45- cells. Lysis caused a significant decrease of CD45- (−85%) and CD45+ (−36%) cells. (B) The forward/sideward scatter diagram revealed an almost complete depletion of erythroid cells (arrow) whereas other main bone marrow populations remained largely unchanged. (C) Quantification of the cell fractions revealed that the loss of CD45+ cells due to lysis occurred symmetrically among the main leukocyte subpopulations. (D) Staining against an erythroid marker supported the finding that most cells of the erythroid line (CD45−/Ery+) disappeared following lysis whereas other CD45−/Ery- cells were significantly enriched. This was further potentiated after the application of Ficoll, Percoll or MACS depletion leading to an extended loss of erythroid cells in favor of CD45−/Ery-. Values are means ± SD for 5 samples. *p<0.05 by t-test.

Rat bone marrow obtained from femurs and tibiae was pooled and yielded 1.2×10E9±2.4×10E8 cells per animal that were composed of CD45+ leukocytes (62.3±3.7%) and CD45- cells (37.8±3.7%; Figure 2A). The latter population primarily belonged to the erythroid lineage (CD45−/Ery+ cells), but also contained a small number of CD45−/Ery- cells (Figure 2D). As intended, short-term incubation of bone marrow cells with lysis buffer caused a 90% decrease of erythroid cells that was accompanied by a significant loss of about 35% of the CD45+ leukocytes (Figure 2A, B). However, we found the leukocyte loss being equally distributed to the major bone marrow subpopulations (Figure 2B, C). Both unprocessed and lysed bone marrow consisted of two-third BMNCs and one-third PMNs. The BMNC fraction was further classified into B-cells (52.1±5.5%), monocytes/dendritic cells (Mo/DC) (5.1±1.4%) and other CD45+ cells (9.4±1.7%; Figure 2C). The small CD45−/Ery- cell population was hardly affected by lysis and increased to approximately 40% of the CD45- cells within lysed bone marrow (Figure 2D). Interestingly, we found that the isolation of BMNCs (irrespectively of the method used; data not shown) resulted in a further enrichment of CD45/Ery double-negative cells alongside with an additional decrease of erythroid cells (Figure 2D).

### Establishing an Adapted Density Separation Gradient using Percoll

**Figure 3:**
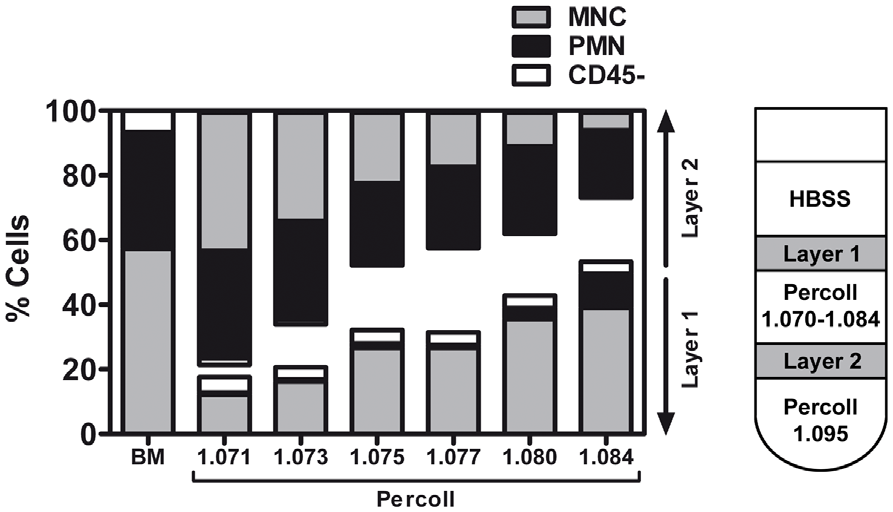
Adjusted density gradient centrifugation by using Percoll. Percoll of different densities (1.071 to 1.084 g/mL) differentially separated the major subclasses of lysed rat bone marrow. The higher the density, the higher was the BMNC yield (within layer 1) and the lower the BMNC loss (within layer 2), respectively. The CD45- population remained stable in layer 1 at densities from 1.073 g/mL upwards. At a density of 1.084 g/mL the BMNCs were increasingly contaminated with PMNs. Values are means from 2 pooled samples per density.

We used a series of different Percoll separation gradients to identify the density gradient most suitable to separate MNC and PMN from rat bone marrow. The main endpoints were BMNC loss and yield as well as PMN contamination. We found a positive relation between escalating Percoll densities and BMNC yield (Figure 3) with highest BMNC yield at densities 1.080 g/mL and 1.084 g/mL. However, at a density of 1.084 g/mL, we observed a distinct increase of PMN contamination (Figure 3).

### Rat BMNC Recovery following Different Isolation Protocols

**Figure 4:**
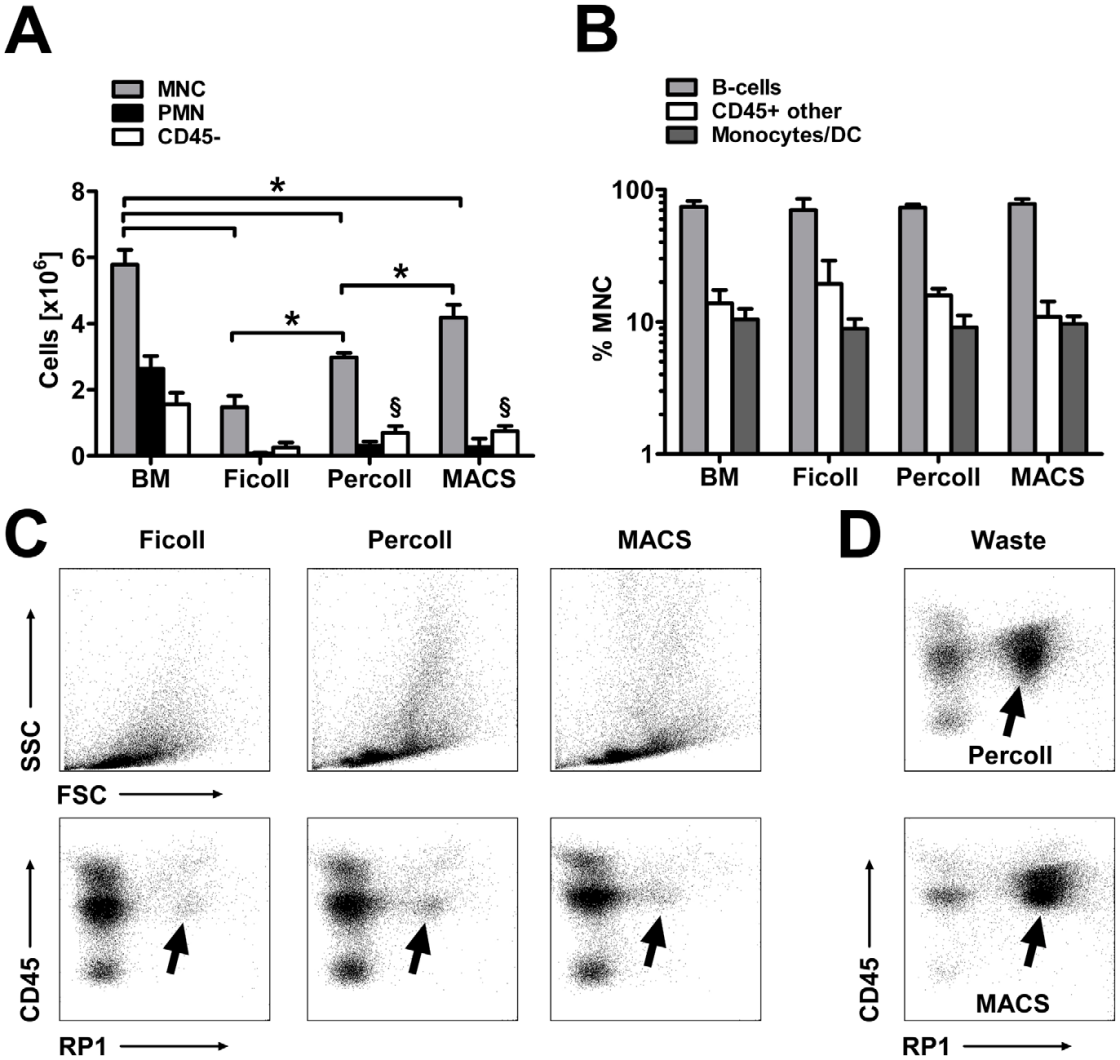
Rat BMNC yield following different isolation procedures. (A) Compared to lysed bone marrow, each of the isolation procedures caused an almost complete depletion of PMNs (C; arrows indicate the remaining RP1+ PMNs). As an unwanted side effect, this was accompanied by a significant loss of BMNCs and CD45- cells (A). BMNC loss was maximal after Ficoll DGC followed by Percoll DGC and MACS separation (*p<0.05). Both Percoll and MACS preserved the CD45- population compared to Ficoll (A; ^§^p<0.05). Further analysis revealed a symmetric cell loss among the BMNC subpopulations (B; C, representative forward/sideward scatter diagrams). (D) For both Percoll and MACS, primarily RP1+ PMNs but also particular BMNC populations were detected within the waste (Percoll: layer 2; MACS: content of the columns). Values are means ± SD for 5 samples. *^,§^p<0.05 by one-way ANOVA.

We next compared BMNC yield and composition after applying either the Percoll gradient selected above (1.080 g/mL), a standard Ficoll gradient or an immunomagnetic depletion (MACS) of PMNs. Pre-isolation, 10E7 lysed bone marrow cell contained about 5.7×10E6 BMNCs, 2.6×10E6 PMNs and 1.7×10E6 CD45- cells (Figure 4A). As expected, all isolation protocols caused a nearly complete depletion of PMNs (Ficoll: 2.5±1.3%, Percoll 11.5±6.1% and MACS 9.5±8.5% recovery of total PMNs in lysed bone marrow) and, to a lesser extent, of the CD45- population, particularly of the erythroid cells (Figure 4A,C). Depletion of the CD45- cell population was significantly more severe after Ficoll DGC compared to Percoll DGC and MACS (14.6±8.3% versus 41.2±5.1% and 51.0±6.0% recovery of total CD45- cells in lysed bone marrow). One major result of this study is the significant decrease of the BMNC population after each of the isolation procedures. This cell diminution, however, was especially pronounced after Ficoll DGC, during which almost 75% of the BMNC were lost. The adjusted Percoll gradient yielded two-fold more BMNC than the Ficoll DGC, but still showed a BMNC loss of about 50%. By contrast, immunomagnetic depletion of PMN showed a BMNC deficit of less than 30% (Figure 4A). Proportions of leukocyte subpopulations within the respective BMNC fractions resembled those of lysed bone marrow (Figure 4B) indicating symmetric BMNC loss independent of the isolation protocol.

To investigate the amount and composition of cell loss after isolation, we analyzed the cells within layer 2 after Percoll DGC and the eluate after MACS. As expected, we detected large amounts of PMNs both after Percoll and MACS separation (61.3±18.0% versus 75.8±16.5% recovery of total PMNs in lysed bone marrow). By contrast, the incidence of BMNCs was significantly increased within the waste after Percoll DGC compared to MACS (10.6±1.2% versus 4.4±2.7% recovery of total BMNCs in lysed bone marrow; Figure 4D).

### Determination of Rat Progenitor Frequencies

**Figure 5:**
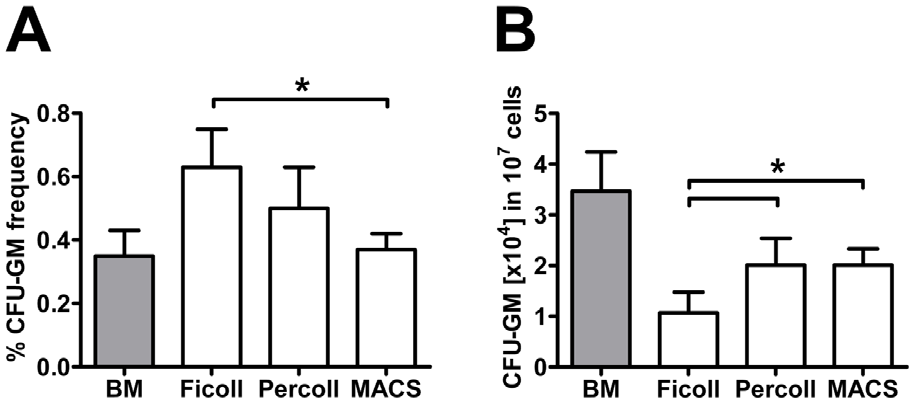
Recovery of rat hematopoietic progenitors. (A) Analysis of the CFU-GM frequency revealed a significant enrichment of hematopoietic stem cells (HSC) by Ficoll compared to MACS. However, when relating the CFU-GM number to the absolute BMNC yield, Ficoll DGC resulted in a significant loss of HSCs compared to both Percoll and MACS separation. Values are means ± SD for 4 samples. *p<0.05 by one-way ANOVA.

Additional experiments were performed to clarify whether or not the different isolation protocols influence the progenitor frequencies within rat BMNCs. To answer this question we used both CFU-GM and CFU-F to assess the proportion of bipotent hematopoietic stem cells (HSC) and of non-hematopoietic progenitors such as mesenchymal stromal cells (MSC). In lysed bone marrow, the CFU-GM frequency was 0.37±0.08% which corresponds to 3.5×10E4 HSCs in 1×10E7 bone marrow cells (Figure 5A, B). Separation of BMNCs by means of Ficoll DGC caused an enrichment of HSCs resulting in a CFU-GM frequency of 0.63±0.12%. This effect was less pronounced after Percoll DGC and disappeared after immunomagnetic separation (0.5±0.13% and 0.37±0.05%; Figure 5A). However, considering the significant different BMNC yields (Figure 4A), only one-third of all HSCs were harvested after Ficoll DGC. By contrast, after adjusted Percoll DGC and immunomagnetic separation, the HSC yield was two-fold higher as compared to Ficoll DGC and increased to two-third of the total amount in lysed bone marrow (Figure 5B). As described previously [29], CFU-F frequency was extremely low in lysed bone marrow (0.00007±0.00002%) representing only 7 of non-hematopoietic progenitors per 1×10E7 bone marrow cells. We did not detect any CFU-F within the BMNCs after Ficoll, Percoll or immunomagnetic separation nor within the discharge of the latter two.

### Isolation of Human BMNCs

**Figure 6:**
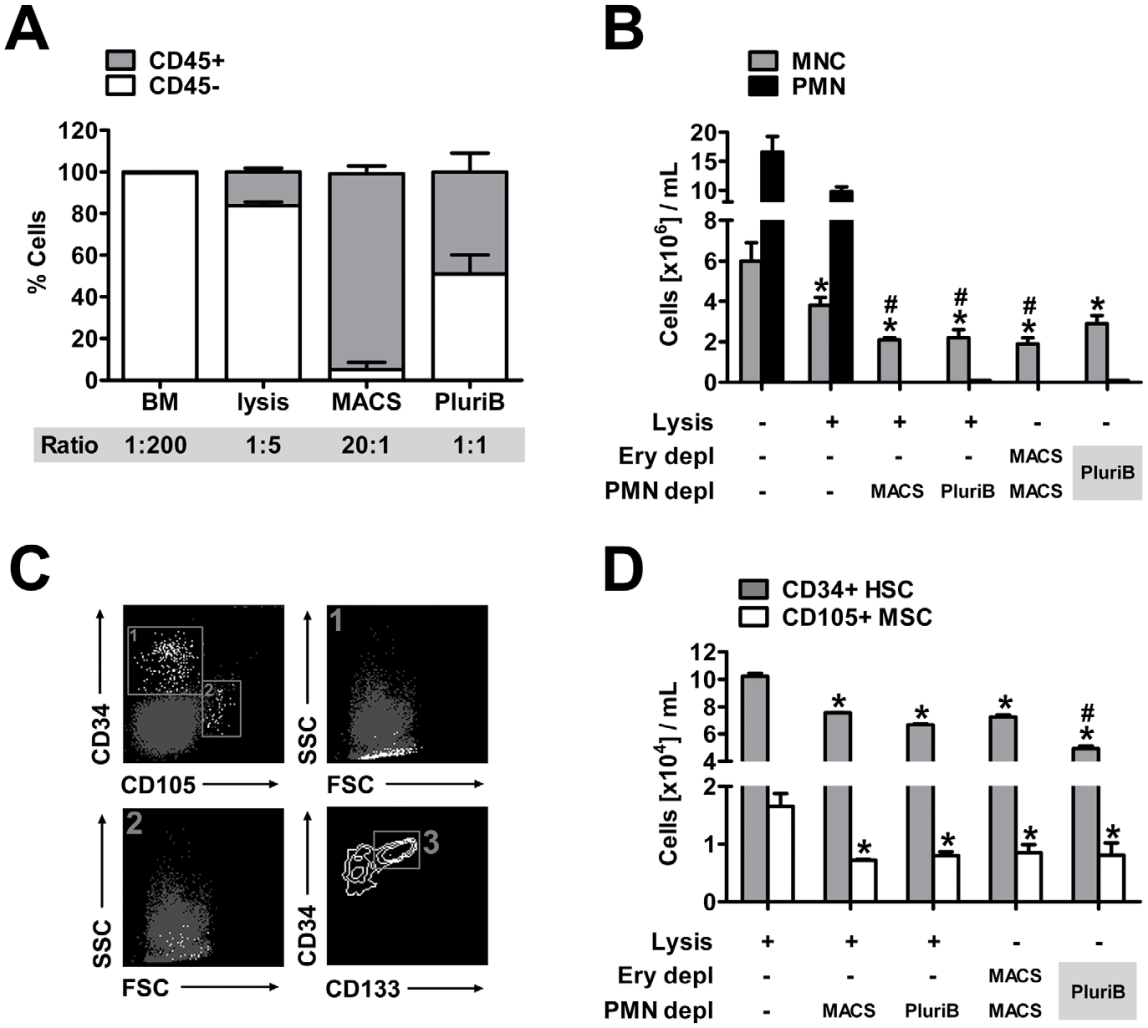
Isolation of BMNCs from fresh human bone marrow aspirates and determination of progenitor cells. (A) Fresh whole bone marrow (BM) contained a high proportion of CD45- erythroid cells. The amount of erythroid cells could be significantly decreased by lysis or by immunodepletion (MACS or PluriB) of erythrocytes whereas highest purity of CD45+ leukocytes was attained after immunomagnetic depletion (MACS). (B) Lysis of whole bone marrow caused a significant loss of BMNCs (*p<0.05 versus whole bone marrow) that was further extended by PMN depletion with either MACS or PluriB (^#^p<0.05 versus lysed bone marrow). The combined depletion of erythrocytes and PMNs by sequential MACS or by combined PluriB yielded comparable BMNC counts. The amount of remaining PMNs was constant at a low level after each of the isolation procedures. (C) Gating strategy for progenitor characterization. CD34+ hematopoietic stem cells (HSCs; C 1) featured a uniform, lymphoid-like phenotype and were partially CD133+ (C 3). In contrast, CD105+ mesenchymal stromal cells (MSC; C 2) exhibited increased variability of size and granularity. (D) Quantification of progenitors revealed that HSCs and MSCs were lost due to the different BMNC isolation procedures (p<0.05 versus lysed bone marrow). Cell loss was comparable in all experimental approaches except for the combined one-step depletion (PluriB), where the HSC yield was significantly lower compared to the other isolation protocols (^#^p<0.05 versus lysis+MACS, lysis+PluriB and MACS+MACS). Values are means ± SD for 3 samples. *^,#^p<0.05 by one-way ANOVA.

To further validate whether the concept of immunodepletion is a feasible method to isolate BMNCs also from human samples, we used four different approaches to deplete both erythrocytes and PMNs of fresh human bone marrow aspirates. After harvest and pretreatment, whole human bone marrow contained 22.6±3.6×10E6 CD45+ leukocytes per mL compared to 4.68×10E9±3×10E8 CD45- cells per mL, compromising predominately CD235+ erythrocytes. Thus, a ratio of leukocytes and erythroid cells of 1∶200 was determined (Figure 6A). Depletion of erythrocytes was hence indispensable for the further flow cytometric characterization and antibody-based processing of human bone marrow. Standard lysis led to a significant decrease of CD45- cells yielding a ratio of leukocytes and erythroid cells of 1∶5. Immunodepletion of erythrocytes by either MACS or pluriBeads (PluriB) was even more effective and resulted in a ratio of 20∶1 and 1∶1, respectively (Figure 6A).

Next, we isolated BMNCs from lysed bone marrow by immunodepletion of CD15+ PMNs using either MACS or PluriB. Both methods were effective to significantly decrease PMN numbers to less than 1% of the content in whole bone marrow and recovered almost 60% of the lysed bone marrow MNCs (Figure 6B). The combined depletion of erythrocytes and PMNs by either MACS (two-step approach) or PluriB (one-step approach) showed a comparable BMNC yield and purity except for a slightly, statistically not significant higher BMNC yield after PluriB depletion (Figure 6B).

### Flow Cytometric Determination of Human Progenitor Cell Populations

In addition to the BMNC yield and the remaining PMN content, we used flow cytometry to quantify progenitor populations within the obtained BMNC populations. Hematopoietic stem cells (HSCs) were identified as CD34+, CD105- and CD45dim cells which appeared along the lymphoid population in the forward/sideward scatter plot (Figure 6C; 1). In contrast, CD105+ and CD45- mesenchymal stromal cells (MSCs) were characterized by increased size and granularity (Figure 6C; 2). The HSC population was further differentiated into CD133- and CD133+ cells (Figure 6C; 3). Overall, the CD133+ HSCs accounted for approximately 40% of the CD34+ cells in all experimental groups (data not shown).

Flow cytometric quantification of HSCs and MSCs was not feasible in human whole bone marrow, owing to the extremely low rate of progenitor cells (approximately 0.005% (HSCs) and 0.0005% (MSCs) of all events in whole bone marrow). However, after lysis of human bone marrow, we ascertained 10.2±0.2×10E4 HSCs and 1.6±0.2×10E4 MSCs per mL (Figure 6D). The subsequent isolation of BMNCs was responsible for a significant loss of both HSCs and MSCs (on average of 30% for HSCs and of 55% for MSCs). The decrease of HSCs was thereby mostly pronounced after one-step depletion of erythrocytes and PMNs using PluriB (Figure 6D). However, the proportion of progenitors to the amount of total nucleated cells (i.e. frequency) increased after BMNC isolation, simply as a consequence of selective PMN depletion. Thus, the HSC frequencies increased from 0.76±0.04% in lysed bone marrow to 3.51±0.16% (lysis+MACS), 2.97±0.05% (lysis+PluriB), 3.8±0.06% (MACS+MACS) and 1.58±0.3% (PluriB combination). The MSC frequencies increased from 0.12±0.007% in lysed bone marrow to 0.34±0.01% (lysis+MACS), 0.37±0.11% (lysis+PluriB), 0.44±0.04% (MACS+MACS) and 0.26±0.04% (PluriB combination).

## Discussion

In this study, we provide evidence that the application of density gradient centrifugation (DGC) for isolation of the mononuclear cell fraction from rat bone marrow (BMNC) is confounded by a significant loss of the cells of interest. When using Ficoll as density medium, only 25% of the BMNCs being detectable in lysed bone marrow were recovered. These findings are consistent with other studies investigating human bone marrow that show BMNC recovery rates between 15 to 30% after Ficoll DGC [17], [19]. Interestingly, we found that DGC using Percoll with an equal density to that of Ficoll (1.084 g/mL) yielded significantly more BMNCs (Figure 3), suggesting that excessive cell loss during Ficoll DGC is a consequence of density medium-related cytotoxicity [30]. Moreover, Ficoll based DGC has been primarily established for separation of blood into its components. In contrast to peripheral blood mononuclear cells (PBMNCs), BMNCs exhibit an increased variability of cell maturity and consequently of buoyant density [31]. Hence, BMNCs with densities deviating from those of PBMNCs might less likely accumulate within the correct Ficoll density layer. To address this problem, we used a series of different layer densities by diluting Percoll and found that the optimal density range for isolating rat BMNCs is in between 1.080 to 1.084 g/mL, bounded above by unwanted polymorphonuclear cell (PMN) contamination and below by decreasing BMNC yields (Figure 3). However, even after adjustment of the density layers, density gradient separation of BMNCs was generally confounded by a cell loss of at least 50%. This is highly relevant for clinical practice, since meta-analyses revealed a clear dose-dependency of autologous BMNC transplantation [20]. A profound cell loss during DGC may hence directly attenuate therapeutic efficacy.

Consequently, we looked for an alternative method to separate BMNCs with a higher yield, and decided to immunomagnetically deplete PMNs as it has been described for human blood samples from patients with sepsis or burn [28]. Immunomagnetic separations by either positive or negative selection of special cell populations can be conducted under conditions of good manufacturing practice [32] and have already been applied in several clinical studies [33]–[35]. In our experiment with rat samples, we show that this approach achieved a significantly higher BMNC yield compared to both DGC-based methods (Figure 4A), while the leukocyte composition was identical. We therefore conclude that immunomagnetic separation could be an appropriate method to isolate bone marrow mononuclear cells for transplantation purposes offering the advantage of higher BMNC doses for patients. To further validate this hypothesis, we performed a proof-of-concept study with fresh human bone marrow samples from three healthy donors. We found that immunomagnetic separation (MACS) of CD15+ PMNs from lysed human bone marrow was feasible and yielded a BMNC recovery of 55%. An alternative method for immunoselection based upon differently sized beads (PluriB) recovered slightly more cells (58%) with comparable effort and almost equal final composition of BMNCs. Generally, human BMNC yields were lower compared to what we observed in our rat study (72%), but we assume that a further optimization of the human proof-of-concept protocol, as it has been extensively done for the rat protocol, would approximate the BMNC yields.

One further important advantage of using immunoselection compared to density gradient centrifugation is the flexibility to adapt the protocol to a supposed mechanism of action by either depletion of adverse or enrichment of beneficial cell populations. We verified this concept in human bone marrow samples by immunoselection of both PMNs and erythrocytes, since it was shown that the content of erythroid cells directly impaired the function of BMNCs [15]. Interestingly, immunomagnetic depletion of erythrocytes resulted in a highly purified CD45+ cell population (ca. 5% erythroid cells; Figure 6A) compared to lysis (ca. 80% erythroid cells) or Ficoll DGC (ca. 70% erythroid cells [15]).

Hematopoietic CD34+ stem cells (HSC) account for approximately 1.5% of the BMNCs and have been discussed to have a crucial impact on the observed therapeutic effects [33]. DGC-based separation of BMNCs was hence often justified to augment these progenitors within the cell graft [27]. In fact, we and others [12] found a significant enrichment of HSCs after Ficoll DGC. An increased resistance against the toxicity of the density medium or a suitable buoyant density (i.e. close to lymphocytes) might explain HSC enrichment after Ficoll DGC. Nevertheless, due to the significant loss of BMNCs, the overall HSC yield was significantly decreased after Ficoll DGC. Enrichment might thus be beneficial in preclinical studies with a fixed, non-autologous BMNC transplantation scheme. However, in a clinical and autologous setting, DGC might rather result in a loss of effector cells. In fact, the amount of CD34+ cells in human whole bone marrow was described to be 1.5×10E5 per mL [36] whereas several clinical trials yielded between 1.4 to 7.2×10E4 CD34+ cells per mL [12], [13]. After immunoselection of PMNs from lysed human bone marrow, we found 7.5×10E4 CD34+ cells per mL which is considerably higher compared to that what was reported recently in large scale clinical studies.

It is still controversially discussed which cell population within BMNCs is actually required for beneficial effects and which is not. Thus, for example, one recent study described the B-cells as being the effective cell population within the BMNCs [37]. In the present study we observed a distinct CD45- cell population within the rat bone marrow cells which clearly did not belong to the erythroid line (Figure 2D). In total the CD45- population was almost completely depleted after Ficoll DGC (15% recovery of total CD45- cells in lysed bone marrow), a finding that is supported by Medina et al. [38] who found many non-erythroid CD45- cells within the usually discarded pellet after DGC. By contrast, in our rat study, both Percoll and MACS separation conserved a large proportion of this population (41% and 57% of lysed bone marrow; Figure 4A). Interestingly, these CD45- cells seem to contain specific stem cell populations such as MSCs, VSEL, epithelial progenitors [38], multilineage inducible cells [39] and endothelial progenitor cells [40] and might thus be relevant for therapeutic effects of BMNCs. In line with these observations, it was recently described that most of the bone marrow MSCs were discarded during Ficoll DGC due to their aggregate nature [23], and that whole bone marrow, but not BMNCs obtained by Ficoll DGC improved functional recovery after experimental myocardial infarction [41]. Collectively, these considerations question whether the concept of stem cell enrichment during Ficoll DGC-based BMNC separation is true and reasonable in the setting of clinical studies.

In conclusion, our findings show that the isolation of BMNCs by density gradient centrifugation causes a distinct and symmetric cell loss that includes a decrease of therapeutically relevant stem cell populations. A higher cell yield can be obtained by using a customized Percoll protocol or by immunodepletion of unwanted constituents such as erythrocytes and granulocytes.
